# The role of melatonin on caspase-3-like activity and expression of the genes involved in programmed cell death (PCD) induced by in vitro salt stress in alfalfa (*Medicago sativa* L.) roots

**DOI:** 10.1186/s40529-022-00348-7

**Published:** 2022-06-11

**Authors:** Shabnam Jalili, Ali Akbar Ehsanpour, Seyed Morteza Javadirad

**Affiliations:** 1 Department of Plant and Animal Biology, Faculty of Biological Science and Technology, University of Isfahan, Isfahan, Iran; 2 Department of Cell and Molecular Biology, Faculty of Biological Science and Technology, University of Isfahan, Isfahan, Iran

**Keywords:** Melatonin, Salt stress, *Medicago sativa*, Apoptosis, *VPE*, *UCP*, *BI-1*

## Abstract

**Background:**

Alfalfa (*Medicago sativa* L.) is the most cultivated forage plant as a model in legumes. Salinity stress due to Na^+^ toxicity causes severe, oxidative stress as a main reason for program cell death (PCD) in plants. Melatonin application can increase plant productivity in response to diverse stressors via modulating plant antioxidant mechanisms and PCD inhibition in plants.

**Results:**

Alfalfa roots were subjected to different concentrations of in vitro salinity supplemented with melatonin (0.1, 10 and 15 µM) for ten days. Application of melatonin under salinity stress reduced ROS, H_2_O_2_ and ${\text{O}}_{2}^{ - }$ content and showed a dramatic impact on TTC reduction and augmented cell viability. Interestingly, melatonin inhibited caspase 3-like protease activity and could decrease DNA fragmentation induced by salinity while increased expression of anti-apoptotic genes *BI-1, UCP1-UCP2* involved in PCD pathway. In contrast, in 300 mM salinity, *γVPE* gene as a proapoptotic of PCD down-regulated significantly.

**Conclusions:**

For the first time, present data showed that, melatonin plays a major function in preventing PCD in alfalfa root meristem cells. We attempted to offer a mechanism for the function of melatonin as an anti-apoptotic agent by demonstrating significant actions of melatonin on mitochondria proteins, such as UCPs, in a manner similar to animal cells.

## Background

Plants are often exposed to several environmental stresses in their habitats. Many plant species develop adaptive mechanisms, in which the cells and tissues tolerate excessive abiotic and biotic stresses and delay programmed cell death (PCD) (Drew et al. 2000; Joseph and Jini 2010). Salt stress is one of the major limiting factors for crop growth and yield. A high concentration of salinity reduces all growth aspects in the shoots and roots (Munns and Tester 2008). Roots are inextricably linked to the ecosystem, in which they develop. Root growth is restricted to a small area of the root axis called root apical meristem, which is very special among root cells. The apical-meristem undergoes mitotic division to generate new cells for root development and nutrient absorption but there has been not much finding on these regions under extreme salt stress (Bitonti and Chiappetta 2010).

PCD is a genetically specified process that occurs during the normal life cycle of the plants, as well as in response to changing environmental conditions. Developmental processes such as leaf morphogenesis and senescence, as well as abiotic or biotic stresses, induce PCD. Also, it is involved in the defense mechanisms of plants (Vardar and Ünal 2011; Wang et al. 2011). Plants and animals have similar biochemical properties when it comes to PCD, such as cell shrinkage, vacuolization, mitochondrial cytochrome c release, stimulation of a particular protease, chromatin aggregation, and the cleavage of genomic DNA into internucleosomal 180-bp segments, a process known as DNA laddering (Papini et al. 2011; Poor et al. 2013). According to various reports, high salinity has induced PCD in plants such as tobacco protoplast (Lin et al. 2006) and soybean root tips (Kosova et al. 2018). The impact of melatonin on PCD and its regulation has been poorly understood. Salt stress, as one of the most common abiotic stressors, generates oxidative stress, resulting in the formation of reactive oxygen species (ROS), and disrupting membrane, and protein, as well as DNA. As a result, ROS can affect a wide range of signal transduction systems, offering beneficial or harmful feedback regulatory mechanisms. As a general rule, smaller levels of ROS are used as an indicator in the stress responses but larger amounts of that may probably induce PCD (Ashraf 2009). Plant cells have developed sophisticated strategies to control intracellular ROS and detoxify high amounts of ROS. One of the most important signals in PCD is hydrogen peroxide (H_2_O_2_) and superoxide radical (${\text{O}}_{2}^{ - }$), which are elevated during exposure to severe stress conditions and induce some cellular reactions such as caspase-like proteases (Raja et al. 2017). Caspase-3 plays a vital role in the regulation of PCD, which belongs to the cysteine-aspartic acid protease family and it is a common active death protease that catalyzes several essential cellular proteins (Van and Lamkanfi 2019). The equilibrium between ROS generation and ROS elimination is critical for avoiding oxidative damage and PCD induced by oxidative stress. Thus, reduced efficiency of ROS scavenging by specific metabolites would be expected to play a major role in the onset of PCD (Hasanuzzaman et al. 2020).

Melatonin (*N*-acetyl-5-methoxytryptamine) is a growth regulator made from serotonin that has been acetylated and methylated (Arno and Hernandez 2015). Melatonin possesses plant growth regulator characteristics or prevents plant leaf aging, which is triggered by stress. Several studies have found that melatonin has ROS scavenging abilities, and along with antioxidant characteristics, can reduce the risk of stress (Khan et al. 2020). Melatonin's antioxidant behavior can be divided into four groups: (a) direct free radical scavenging; (b) stimulation of activities of antioxidant enzymes; (c) augmentation of the efficiency of mitochondrial oxidative phosphorylation and reduction of electron leakage; (d) enhancement of the efficiency of other antioxidants’ metabolites such as ascorbate and glutathione, which are important components of the antioxidant system, such as vitamins C, E and intermediate product of melatonin with the high ability for radical scavenging (Michard and Simon 2020; Nawaz et al. 2021). Melatonin triggers a free radical scavenging cascade that proceeds cell defense, making it very efficient in preserving cells from oxidative stress even at low content. Recently several studies have shown that melatonin improves the antioxidant system and reduces ROS accumulation in *Lolium perenne* during dark treatment (Zhang et al. 2016), watermelon under salt stress (Li et al. 2017), and cucumber under chilling stress (Marta et al. 2016). After 1994, when the first study describing the function of melatonin in apoptosis in animals was introduced, the number of researches in this field has been increased; however, only two studies on carrot suspension cells under cold stress (Lei et al. 2004) and *Nicotiana tabacum* L. line Bright Yellow (BY-2) suspension cells under lead (Pb) stress (Kobylinska et al. 2017) have been reported. Also, melatonin has been reported as an important anti-apoptotic agent in a variety of cancers by reducing cell calcium uptake, eliminating the cytoplasm from the free radical oxidation that can be generated by the mitochondria, and decreasing pro-apoptotic proteins, such as Bax (Tan et al. 2016). It has also been reported that the application of melatonin was able to reverse the apoptotic procedure, reduce DNA fragmentation and Bax (pro-apoptotic protein) content, and decrease the activity of caspases-9 and 3 in human cancer cells (Ferreira et al. 2010). Bax inhibitor-1 (BI-1) is the endogenous cell death repressor, and core regulator of PCD, located in the endoplasmic reticulum (ER) in both animal and plant cells, which modify the content of Ca^2+^ that can be released from the ER (Huckelhoven 2003). Melatonin application blocked Bax pro-apoptotic activity via the SIRT1/NF-kB axis with significant inhibition of cytochrome *c* release in the rat (Sun et al. 2015). BI-1 is essential for normal plant growth; however, it plays a defensive role against both biotic and abiotic stresses (Zheng et al. 2007). New studies have presented vacuole rupture-mediated cell death and nuclear degradation during PCD in plants. As initiators of plant PCD, vacuolar processing enzymes (VPEs) are cysteine proteinases involved in cell death through vacuolar collapse (Hara-Nishimura and Hatsugai 2011). VPEs and caspase-1 share conserved structural properties but VPEs localize in vacuoles, while animal caspases localize in the cytosol (Lu et al. 2016). Kuroyanagi (2005) reported that there are four *VPE* in the genome of *Arabidopsis thaliana*. The expression of *αVPE* and *γVPE* has been discovered in vegetative organs, and *γVPE* recognizes both *VPE* substrate with Km of 30.3 µM and a caspase-1 substrate with Km of 44.2 µM. As a result, *γVPE* will show caspase-1-like activity (Kuroyanagi et al. 2002; Hara-Nishimura et al. 2011). Consequently, it can be assumed that melatonin has a beneficial impact on *γVPE* in plant apoptosis pathways. Based on the studies in humans and animals, melatonin preserves mitochondrial membrane potential and functions by removing ROS, prohibiting the mitochondrial permeability transition pore (MPTP), and activating uncoupling proteins (UCPs). Melatonin by affecting on UCPs balances the membrane potential, increasing electron transport and eventually decreasing ROS generation and oxidative damage in mitochondria (Akhmedov et al. 2015; Vitale et al. 2016); so melatonin, by increasing the expression and activity of *UCPs* can inhibit PCD in plant cells.

Alfalfa is a perennial plant, which grows as forage in the world. This plant has high economical and agronomical significance because of its high forage quality and N_2_ fixation ability in the roots (Anower et al. 2013). Compared with many other crops, *Medicago. sativa* cv. Isfahani is relatively tolerant to salt stress (Ashrafi et al. 2014), although soil salinity is still a significant environmental factor limiting yield in alfalfa. Since melatonin has a significant impact on decreasing animals’ PCD, we hypothesized that melatonin treatment could prevent PCD and improve salt tolerance in alfalfa roots. To verify the hypothesis, we investigated the impact of various melatonin concentrations on two important targets: (1) regulation of ROS generation including H_2_O_2_ and ${\text{O}}_{2}^{ - }$, and (2) DNA laddering, caspase-3-like activity and expression of apoptotic genes such as *BI-1, γVPE, UCP1,* and *UCP2* under salt stress in alfalfa root meristem under in vitro culture.

## Materials and methods

### Plant materials and culture conditions

Seeds of *Medicago sativa* cv. Isfahani from the Pakan Bazr Company in Isfahan, Iran, were used in this research. Alfalfa seeds were surface-sterilized for 1 min with 70% (v/v) ethanol, followed by bleach for 20 min and finally washed three times using sterile distilled water. Seeds were germinated in sterile MS (Murashige and Skoog 1962) medium containing 30 g/l sucrose, 8 g/l agar (pH of 5.8). Seedlings were then transferred to the culture medium enriched with various concentrations of melatonin and salt. All cultures (Maximum 12 repetitions for each treatment) were transferred to the culture room (25 ± 1 °C under 16/8 h (44 μmol phot m^−2^ s^−1^) light/dark photoperiod.

### Experimental designs

To optimize melatonin and NaCl concentrations, we looked at several filter-sterilized (Solarbio life sciences, Beijing, china) melatonin (0, 0.01, 0.1, 1, 10, 15, 30, 50 and 100 µM) and 0, 100, 150, 200 and 250, 300 and 500 mM NaCl, and time courses of 5, 10 and 15 days (unpublished data). High amounts of melatonin (30, 50, 100 M) had no positive effect on neither root growth nor salt tolerance. Based on our data, optimum duration for melatonin impact on root growth was 10 days. Finally, we used 0.1, 10 and 15 µM melatonin treatment and, high salinity concentrations (150, 300, and 500 mM NaCl) were used for PCD analyzing of alfalfa root meristem and 0, 150, and 200 mM NaCl for biochemical tests on roots. Medium without melatonin and NaCl used as control.

### Quantification of H_2_O_2_

The H_2_O_2_ content of alfalfa root was determined using Velikova et al. (2000) method with minor modifications. Approximately, 50 mg fresh roots were ground with 2 ml of 0.1% (w/v) trichloroaceticacid (TCA) and then homogenates were centrifuged at 10,000 rpm for 15 min at 4 °C. Afterward, 0.5 ml of the supernatant was added to 0.5 ml of 10 mM potassium phosphate buffer (pH 7.0) with 1 ml of 1 M potassium iodide (KI). A multi-mode reader was used to measure absorbance at 390 nm. The H_2_O_2_ content was determined by extinction coefficient (0.28 µM^–1^ cm^–1^) and results were expressed as μmol g^−1 ^FW.

### Quantification of ${\text{O}}_{2}^{ - }$

Nitroblue tetrazolium (NBT) staining was used to measure superoxide ion ${\text{O}}_{2}^{ - }$ accumulations. Roots were incubated in 50 mM phosphate buffer (pH 6.4) containing 0.1% NBT and 10 mM sodium azid. Samples were shaken in darkness for 12 h (80 rpm/min). At the site of NBT precipitation, blue formazan compounds were visible. The ${\text{O}}_{2}^{ - }$ content was calculated using a slightly modified approach of Rook et al. (1985).

### ROS measurement

According to Mahalingam et al. (2006), cellular ROS levels are determined when 2′,7′-dichlorofluorescin diacetate (DCFDA) is oxidized and converted to the fluorescence dye 2′,7′-dichlorofluorescein (DCF). The fluorescence generated is directly proportional to the amount of oxidized DCFDA to DCF. About 100 mg fresh roots were homogenized in an ice bath with 10 mM Tris–Hcl (pH: 7.2). The homogenates were centrifuged at 14,000*g* for 20 min at 4 °C. Then 950 µl supernatant was added to 50 µl of 1 mM DCFDA and then vortexed vigorously and incubated for 15 min in the dark. The fluorescence of the samples was measured on excitation/emission wavelength: 480/520 nm.

### Triphenyl tetrazolium chloride (TTC) assay

Approximately 200 mg fresh roots tip immersed in 2 ml incubation solution containing 50 mM potassium phosphate buffer (pH 7.5) and 0.6% triphenyl tetrazolium chloride (TTC) and incubated for 24 h at 30 °C. The formazan generated from living tissue was extracted with 3 ml ethanol at 60 ºC for 30 min and then absorbance was measured at 485 nm using a multi-mode reader. Boiled roots in water were used as the control. Viability was reported as A485 mg^–1^ FW (Ishikawa 1995).

### DNA extraction and analysis

DNA was isolated using Doyle and Doyle (1987) and Cullings et al. (1992) methods with minor modifications. Root tips (0.5 g) with or without salt were ground with 500 µl of 0.2% Cetyl trimethylammonium bromide (CTAB) buffer and 20 µl β-mercaptoethanol. Samples were incubated at 55 °C for 30 min followed by adding 500 µl of 24:1 (v/v) chloroform: isoamyl alcohol, eventually DNA was precipitated with cold isopropanol. Pellets were washed with cold 70% Ethanol and then samples were solved with 100 µl of deionized water for 30 min at 55 °C. Finally, 8 ng of extracted genomic DNA was loaded on 1.5% agarose gels and after the electrophoresis was completed gel image was taken.

### Caspase-3-like activity

To investigate the effects of melatonin on cell death, caspase3-like proteases was measured by Caspase-3/CPP32 Fluorometric Assay Kit. About 0.3 g root meristem was homogenized using 100 mM sodium phosphate buffer (pH: 7.8) containing 2% polyvinylpyrrolidone (PVP), 1 mM ethylene-diamine-tetraacetic acid (EDTA) and 4 mM dithiothreitol (DTT). Then, mixture was centrifuged at 16,000 rpm for 15 min in 4 °C followed by manufacturer’s instruction (Caspase-3/CPP32 2 Fluorometric Assay Kit, Biovision, USA). The caspase-3-like activity was measured by cleavage fluorogenic caspase-3-like substrate DEVD-AFC. The cleavage of DEVD-AFC was continuously observed at 405 nm absorbance by releasing of free fluorescent.

### Real-time quantitative PCR (qPCR)

Total RNA was extracted from the root meristem using TRIZOL reagent according to the manufacturer’s instructions (Yekta Tajhiz Azma, Tehran, Iran). DNase I kit (Thermo Fisher Scientific, USA) was used for removing contaminated DNA from RNA samples. The cDNA was synthesized from 1 µg of total RNA using Kit (Yekta Tajhiz Azma, Tehran, Iran) in 20 µl of reaction mixture. For qPCR reaction mix containing primer pairs (*BI-1, γVPE* and *UCP1-UCP2*) (Table 1) followed by 95 °C for 15 min, 40 cycles of 95 °C for 30 s, 60 °C for 30 s, 72 °C for 30 s according to the protocol of RealQ Plus Master Mix Green (Amplicon, Denmark) using the DNA Engine Peltier Thermal Cycler (BIO-RAD, USA). Each sample was analyzed in three replicates, and relative expression was normalized by 18s RNA as control gene (Livak and Schmittgen 2001).

**Table 1:** The specific primers of genes related to melatonin and apoptosis in alfalfa roots meristem

Primer name	Gene ID	Primer sequence (5′–3′)
*BI-1*	LOC11436967	F: CTTCTGCCATCTTTGGAGGTTCA
		R: CCTTCTCAACTATTTCCTGCGTGT
*γVPE*	LOC11432331	F: TTTCCTCCGATTACCCTCCCAAG
		R: TCCAGTAGCCATTAGAACCAGCAA
*UCP1-UCP2*	LOC25498702	F: GGTCAAGTCGAGAATGATGGGAGA
	LOC25490758	R: GTTCCAAGATCCTAGCCGTCCA

### Statistical analysis

All experiments were carried out in a complete random design with 10 replicates The differences between mean data was analyzed using (ANOVA) followed by Duncan multiple range test using SPSS program, and data were reported as means ± SD (P < 0.05).

## Results

**Fig. 1:**
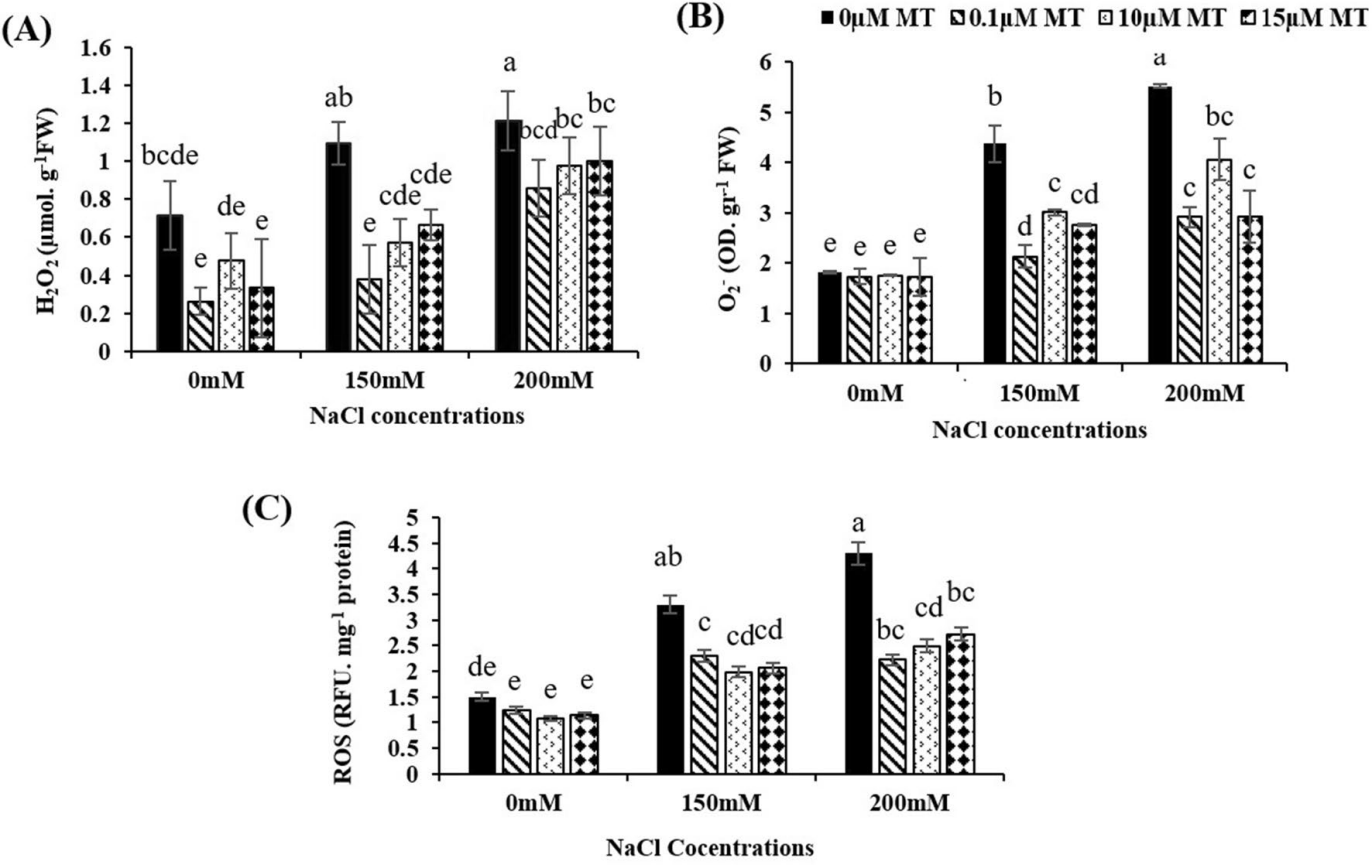
Effects of melatonin (0, 0.1, 10, and 15 µM) and NaCl (150 and 200 mM) on H_2_O_2_ (**A**), ${\text{O}}_{2}^{ - }$ (**B**) and ROS (**C**) content of alfalfa root. Data was collected after ten days of treatment with salt and melatonin. Data represents the mean of replicates ± SD and uncommon letters indicate significant differences based on Duncan's multiple range test (P < 0.05)

Under oxidative stress, the levels of H_2_O_2_ and ${\text{O}}_{2}^{ - }$ are indicators of the root's capacity to scavenge ROS. As shown in Fig. 1, ROS, ${\text{O}}_{2}^{ - }$ and H_2_O_2_ content were increased by NaCl stress. There were no obvious significant differences between the control and the melatonin-treated without salt stress. However, melatonin treatment significantly reduced NaCl-induced accumulation of ROS, ${\text{O}}_{2}^{ - }$ and H_2_O_2_. Under 200 mM NaCl, the content of H_2_O_2_ was reduced by 50%, 41% and 39% (Fig. 1A) whereas about ${\text{O}}_{2}^{ - }$ content deacresed 47%, 26% and 47% (Fig. 1B) and ROS level reduced 48%, 42% and 36%,respectivly, at 0.1, 10 and 15 µM mlatonin concentrations (Fig. 1C).

### The effect of melatonin on TTC reduction

**Fig. 2:**
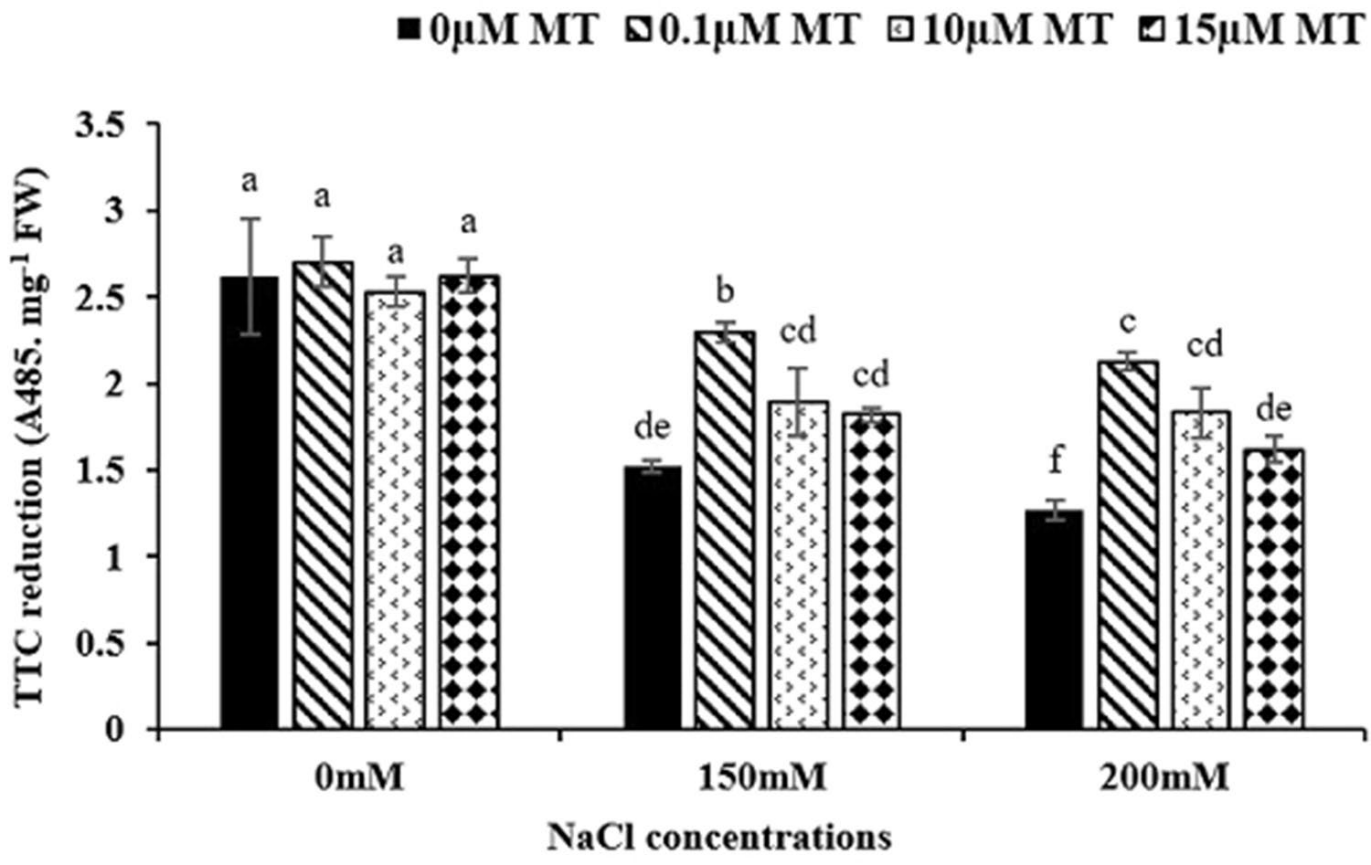
Effects of melatonin and salt pretreatment on root viability in alfalfa roots. Data represents the mean of replicates ± SD and uncommon letters indicate significant differences based on Duncan's multiple range test (P < 0.05)

Cell viability of alfalfa root meristem by measuring TTC is illustrated in Fig. 4. Mitochondrial activity in living cell was evaluated by reduction of Formazan and production of red color. Results indicated that, salt stress (150 and 200 mM) without melatonin decreased cell viability but, melatonin increased cell viability significantly. Under salinity, 0.1 µM melatonin increased viability of the root meristem cells by 51% and 67% compared to the control (Fig. 2).

### Detection of DNA degradation

**Fig. 3:**
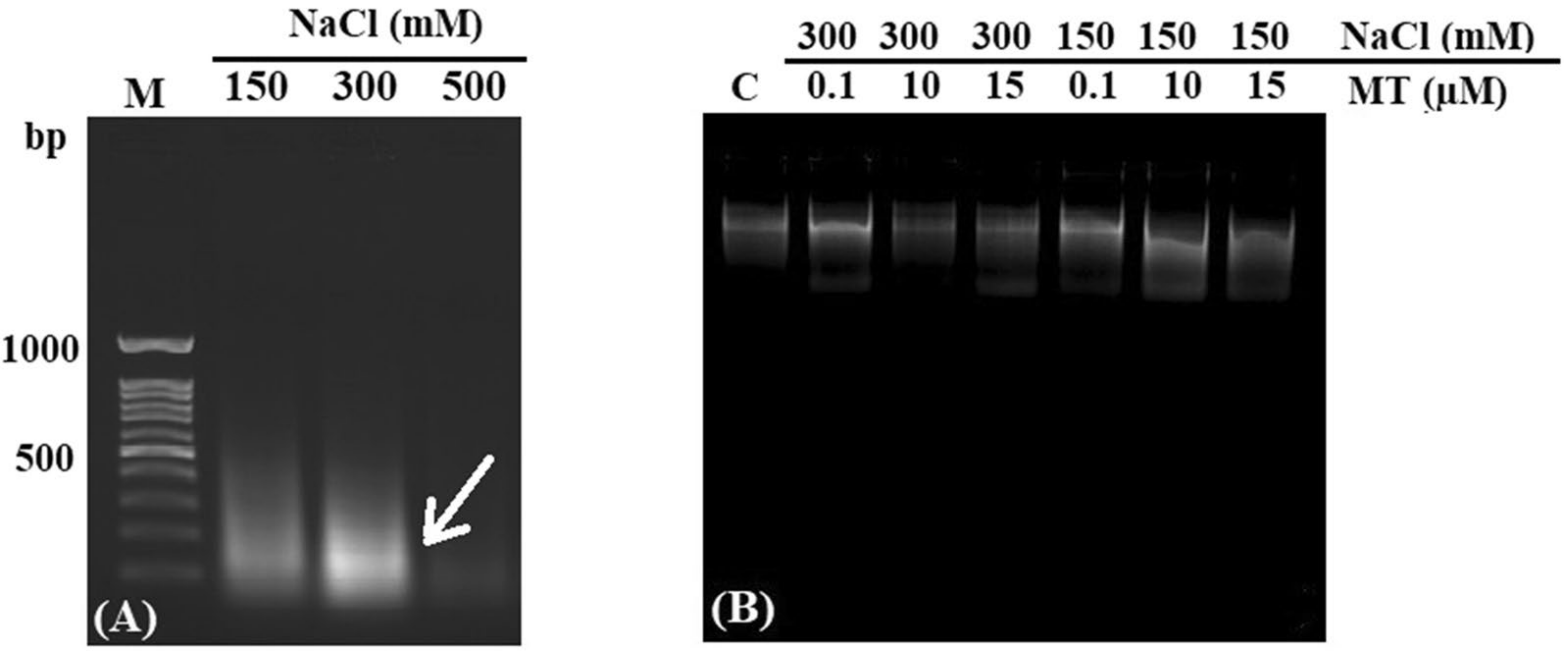
DNA degradation in salt-stressed alfalfa root meristem. Arrows indicate smear and DNA Laddering (**A**). Inhibition of DNA laddering after treatment with melatonin (0.1, 10 and 15 µM) under 150 and 300 mM NaCl (**B**)

To identify DNA degradation as criteria for PCD in root tips, we used a rapid and improved method of DNA laddering assay. Roots meristem were subjected to different concentrations of salt (150, 300 and 500 mM) and thus smear patterns of DNA were displayed (Fig. 3A). Melatonin treatment (0.1, 10 and 15 µM) revealed a clear band on the electrophoresis gel (Fig. 3B). There was no band on the electrophoresis gel under 500 mM NaCl indicating complete destruction of DNA.

### The effect of melatonin on caspases-3-like activity

**Fig. 4:**
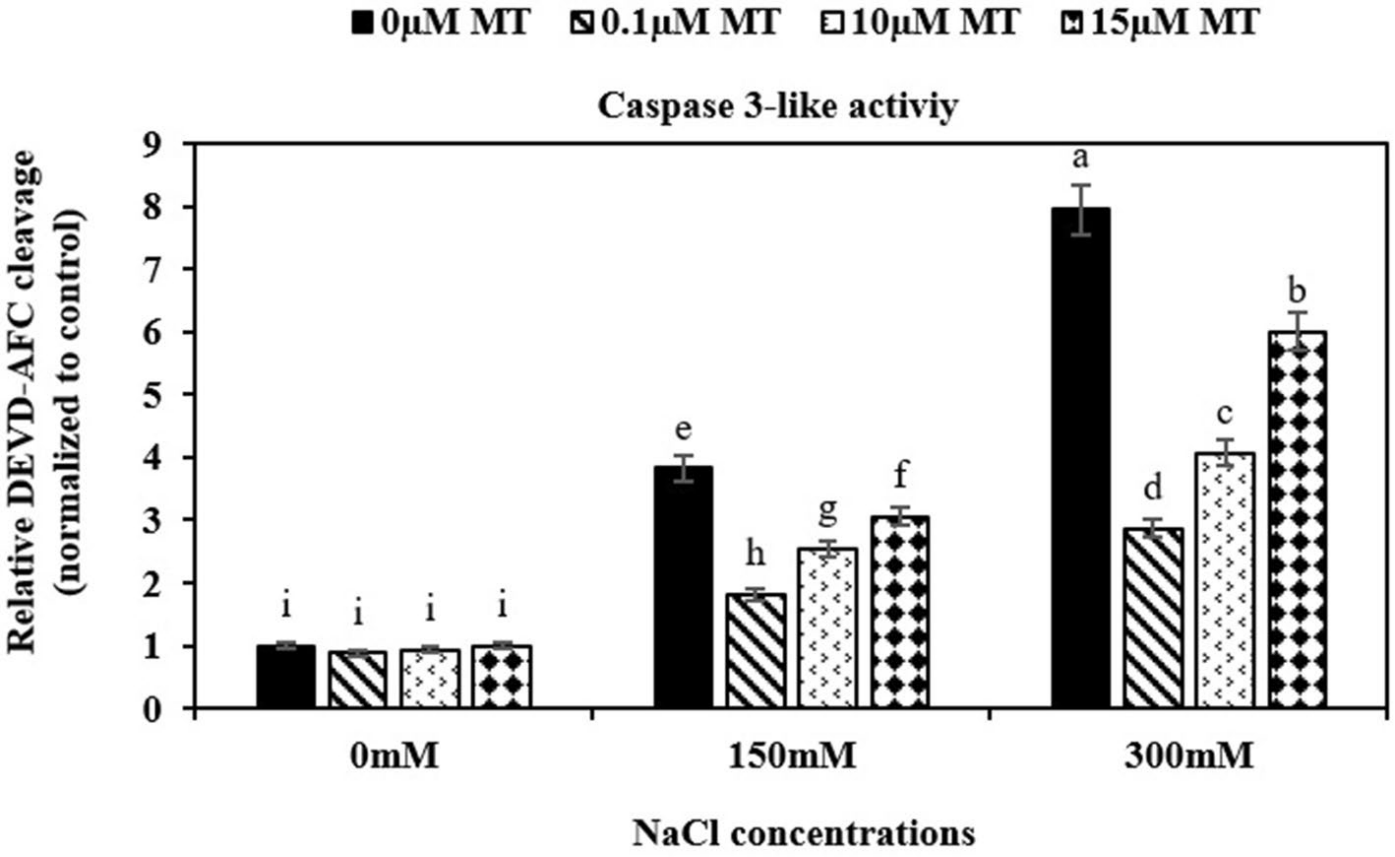
Effect of melatonin on the activity of caspase-3 like proteases based on the cleavage of the substrate, DEVD-AFC. Data represents the mean of replicates ± SD and uncommon letters indicate significant differences based on Duncan's multiple range test (P < 0.05)

Caspase-3 activity was significantly increased in treated root tips with salinity compared to the control (no salinity). When roots subjected to both salt stress and melatonin Caspase-3 activity was decreased. However, increased caspase-3-like activity was observed by increasing of melatonin concentration. Under 150 and 300 mM salility, 0.1 µM melatonin showed the lowest caspase-3 activity (Fig. 4).

### Expression of genes involved in PCD

**Fig. 5:**
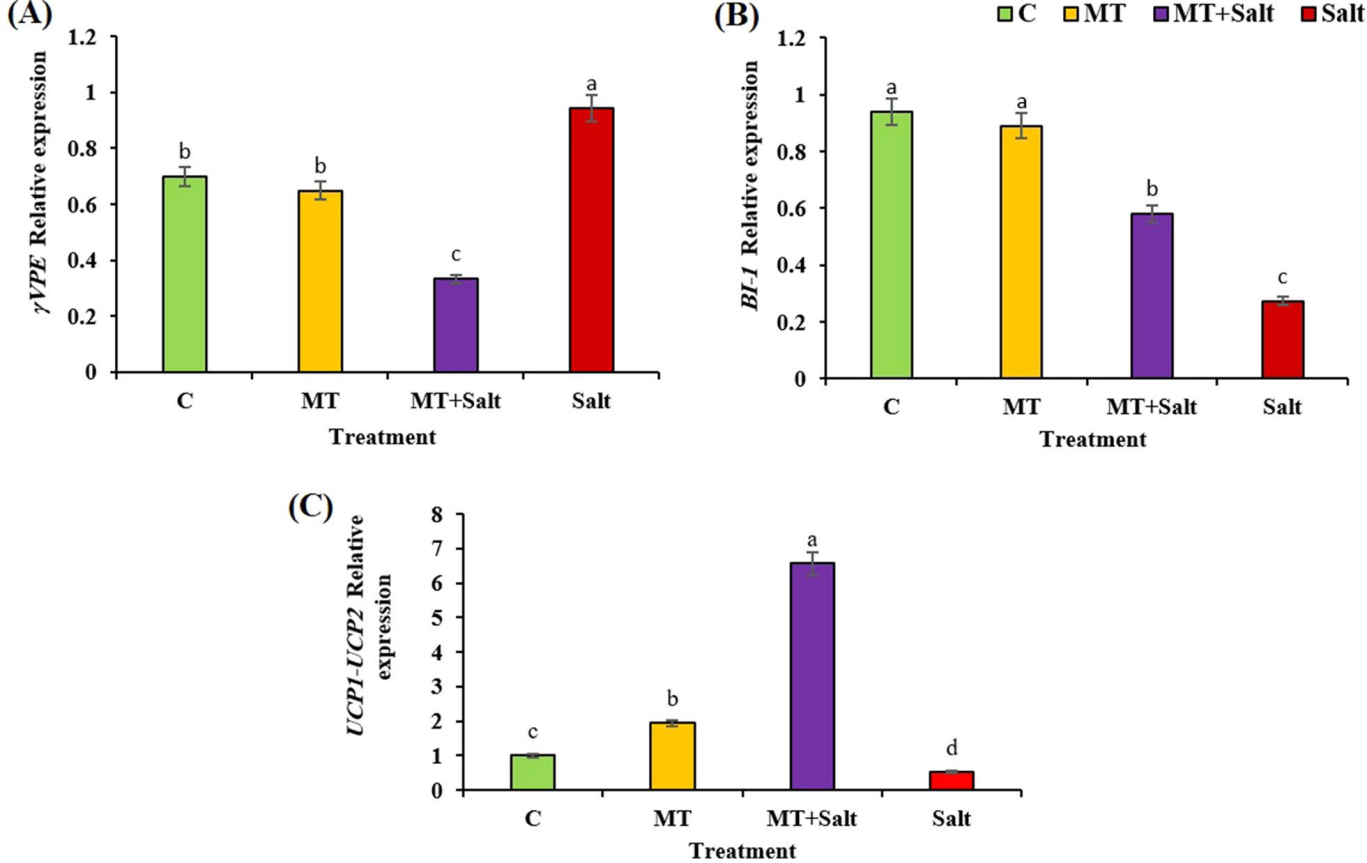
Relative expression of *γVPE* (**A**), *BI-1*(**B**), and *UCP1-UCP2* (**C**) genes in alfalfa root meristem after ten days’ treatment with salt and melatonin. Data represents the mean of replicates ± SD and uncommon letters indicate significant difference based on Duncan's multiple range test (P < 0.05). **C** Control; MT, 0.1 µM melatonin treatment; Salt, 300 mM NaCl treatment; MT + Salt, 0.1 µM melatonin and 300 mM NaCl treatment

To look at the expression of the critical genes involved in PCD pathway in alfalfa roots treated by melatonin the expression pattern of *BI-1, γVPE*, *UCP1* and *UCP2* genes were studied using qRT-PCR. Based on the preliminary experiments and data illustrated in Fig. 5 and Table 1, we selected treated roots with 300 mM NaCl supplemented with 0.1 µM melatonin. In alfalfa root meristem, salinity increased expression of *γVPE* gene whereas, melatonin significantly reduced *γVPE* expression by 66% (Fig. 5A). In contrast, the expression of *BI-1* gene was significantly up-regulated by melatonin + 300 mM NaCl compared to the treated roots with 300 mM salt (Fig. 5B). We investigated the expression of *UCP1* and *UCP2* genes in one primer. Under 300 mM salt, the relative expression *UCP1-UCP2* genes decreased considerably. In contrast roots treated by 0.1 µM melatonin and 300 mM salt dramatically increased 12-fold compared to the roots treated with 300 mM NaCl. Based on our data, Melatonin had the greatest influence on gene expression of *UCP1* and *UCP2* under stress condition (Fig. 5C).

## Discussion

Salinity induces ROS generation leading to PCD and endangers plant cell viability, in order to avoid PCD plant responses to stress by reducing ROS levels (Petrov et al. 2015; Joseph and Jini 2010). This is typically accomplished by tightly controlled antioxidant mechanisms. Several investigations reported that under environmental stress, melatonin has antioxidant properties in plants (Zhang et al. 2015). The anti-apoptotic impact of melatonin on caspase-3 like activity, DNA fragmentation, and expression of the genes involved in PCD in one hand, and induction of antioxidant systems for scavenging ROS on the other hand, creates a valuable mechanism to reduce PCD for the increase of growth and development in alfalfa roots, in salt-stress conditions.

To evaluate whether melatonin could decrease ROS accumulation under high salinity stress, the content of H_2_O_2_, ${\text{O}}_{2}^{ - }$ and ROS was measured. Under high salinity, 0.1 µM of melatonin was more effective than other concentrations in reducing ROS, H_2_O_2_, and ${\text{O}}_{2}^{ - }$ in alfalfa roots. The activation of the antioxidant defense system may be connected to the lowering of these factors by melatonin administration. Increased ROS generation during environmental stress causes lipid peroxidation, protein denaturation, nucleic acid degradation, enzyme inhibition, and the stimulation of the PCD mechanism (Khan et al. 2020). Melatonin has been suggested as a mitochondrial antioxidant because it not only scavenges and neutralizes free radicals directly but also decreases radical generation in mitochondria (Tan et al. 2002). As well, melatonin accelerates electron flux through the Electron Transport Chain (ETC) and slightly inhibits the MPTP to achieve radical avoidance. This may explain why melatonin is more protective for mitochondria against oxidative stress than other antioxidants (Tan et al. 2012). Our results are consistent with those reported by other authors (Shi et al. 2015; Wei et al. 2015). According to the previous studies, melatonin scavenges ROS in *Vigna radiata* leaves and preserves *Gentiana macrophylla* protoplasts from UV-B radiation (Tan et al. 2012). The antioxidant role of melatonin can be interpreted in terms of fine-tuning the concentration of ROS, which is required in the redox regulation of the cell cycle and PCD (Regine Kahl et al. 2004).

The reduction of triphenyl tetrazolium chloride (TTC) is carried out by living tissue, causing the formation of insoluble triphenyl formazan (TF) in red color, indicating the mitochondrial respiratory chain activity (Robert 1951). In our study, only live tissues converted TTC to TF, and as a result, the root was the first plant organ facing the salinity stress; therefore, 200 mM NaCl treatment had the minimum TTC reduction, and 0.1 µM melatonin under salinity stress showed the highest level of TTC reduction. A low concentration of melatonin under 200 mM NaCl by regulating the electron transport chain, lowers the amount of ROS generated in mitochondria via antioxidant enzymes or increases the activity of hydrogenase enzymes that might be responsible for TTC reduction. Castañares et al. (2019) presented similar results where salinity significantly reduced root viability, while 50 µmol/L melatonin improved the viability of cucumber (Zhang et al. 2013), tomato (Liu et al. 2015), and rice (Han et al. 2017). It can be speculated that melatonin with its antioxidant property suppresses oxidative stress and ROS induced by salinity, resulting in increased meristem cell viability.

Salinity stress caused an oxidative burst in the root cells of alfalfa by generating ROS, which resulted in cell death and DNA fragmentation. DNA laddering is a biochemical phenomenon in the cell, leading to cell death (Wyllie 1980). Therefore, detection of DNA laddering is important to understand the mechanisms of PCD in root meristem under salt stress. Our data revealed a clear DNA smeared pattern as a sign of PCD under salinity stress (150, 300, and 500 mM NaCl) in alfalfa root meristem but melatonin reduced DNA fragmentation. Cells in the root apical meristem are hypersensitive to high salinity stress and lead to DNA damage, inducing PCD. Lei reported that DNA isolated from untreated cells with stress was significantly fragmented while DNA from melatonin-treated cells remained unchanged under stress conditions (Lei et al. 2004). Since melatonin has well-known antioxidant and anti-apoptotic properties, it may stimulate DNA repair systems, such as DNA repairing enzymes or expression of the genes involved in DNA integrity, or may induce the system by inhibiting caspase-3-like activity (Reiter et al. 1998).

A series of catalytically active proteins promote PCD machinery when plants are subjected to stress. One of the biochemical characteristics of PCD is the increase of caspase-3-like activities (Shalini et al. 2015). The mechanism through which melatonin inhibits PCD in plants is still unknown. We hypothesized that melatonin somehow interacts with PCD-related enzymes and avoids that by inhibiting the function of the caspase-3 enzyme. Our findings revealed that melatonin at 0.1 µM concentration was the most effective concentration for reducing caspase-3-like activity in root meristem cells, under salt stress. Melatonin may induce a caspase signaling cascade, where caspase-3, 8, and 9 activities were recorded in root tip cells of *Triticum aestivum,* treated with Al_2_O_3_ (Yanık et al. 2017). Melatonin receptors inhibit many signal transduction cascades involved in PCD, such as cyclic adenosine monophosphate (cAMP) synthesis, calcium mobilization, and diacylglycerol synthesis in animals. However, there are insufficient reports on the activity of melatonin in plants (Chan et al. 2002; Kunduzova et al. 2003). As melatonin has an anti-apoptotic impact in mammals and the role of caspase-like proteases in the plant is presumably similar to that of caspase enzymes in mammals, it seemed that melatonin inhibited the apoptosis pathway by reducing caspase-3 like proteases activity and DNA laddering in the alfalfa root meristem.

*BI-1* is an anti-cell death gene in plants and animal genomes (Xu and Reed 1998). Previous research demonstrated that the overexpression of anti-apoptosis genes reduces apoptosis induced by various biotic and abiotic stresses and ameliorates susceptibility stress (Petrov et al. 2015). One candidate regulator for the PCD mechanism in the plant is *BI-1*, a highly conserved, endoplasmic reticulum protein (Xu et al. 2008). The methods by which BI-1 protein inhibited PCD in plants are not fully understood; however, our data showed that melatonin increased expression of *BI-1* under high salinity (300 mM) in alfalfa root meristem cells. Eichmann (2004) reported that overexpression of *BI-1* enhanced barley tolerance to plant pathogens and abiotic stresses, such as heat and drought stress in tobacco and Arabidopsis plants (Isbat et al. 2009; Watanabe and Lam 2006). BI-1 protein possibly inhibits PCD by modulating Ca^2+^ release and/or release of cytochrome *c* from mitochondria (Isbat et al. 2009). Melatonin may have suppressed Bax as a pro-apoptotic protein on the mitochondrial membrane by influencing the relative expression of *BI-1* and changing Ca^2+^ homeostasis, as well as preventing the cytochrome c release, caspase enzyme activity, and DNA laddering during high salt stress in alfalfa root. VPE has been found to have comparable enzymatic characteristics to caspase that triggers PCD. It causes vacuolar disruption and initiates the proteolytic process that leads to PCD (Yamada et al. 2020). Several forms of VPEs have been implicated in PCD so far, including *NbVPE1a* and *NbVPE1b* in Nicotiana and *AtγVPE* in Arabidopsis. In Arabidopsis leaves, FB1 (fumonisin B1)-induced cell death and *γVPE* are the most significant of the four VPE homologs that reduced PCD (Yamada et al. 2020). The goal of this research was to assess and clarify the potential roles of *γVPE* in salinity-induced PCD in the alfalfa root meristem. In the current study, the *γVPE* expression was increased in root meristem due to high salinity; whereas, melatonin treatment reduced the *γVPE* expression and inhibited PCD significantly (threefold).

UCP activation normally has a positive impact on mitochondrial function. Because the root is a non-photosynthetic tissue, thus mitochondria are the main organ of ROS production, playing a major role in induced PCD under severe salinity stress. Under stress conditions, mitochondria reduce ROS by decoupling the electrochemical gradient from the ATP synthesis complex (Barreto et al. 2020). Melatonin inhibited PCD in meristem cells, either by overexpression of *UCP* or promoting UCP activation. It can be speculated that UCP proteins return mitochondrial intermembrane protons to the matrix and reduce membrane potential (∆ψ); as a result, electron leakage and ROS formation are greatly reduced (Tan et al. 2016; He et al. 2016). Here, for the first time, we investigated the influence of melatonin on *UCP1* and *UCP2*, due to its impact on apoptosis pathways in animals. Based on our data, salinity without melatonin reduced the expression of *UCP1* and *UCP2,* while melatonin increased the expression of these genes during salt stress. Interestingly, melatonin showed the best performance in the expression of *UCP1* and *UCP2* in meristem cells, treated with salinity. Melatonin, like animal cells, might affect mitochondria on the PCD pathway by increasing ROS production during PCD and suppressing that by upregulating anti-apoptotic and downregulating pro-apoptotic genes. However, we tried to provide a route concerning the action of melatonin as an anti-apoptotic molecule in root meristem cells and displayed the effects of melatonin on mitochondria in specific, on UCPs protein, much like in animal cells. Future studies should concentrate on determining how melatonin and related genes impact the PCD pathway. Since melatonin has a positive effect on the plant, using this component as salinity protection in agriculture might be useful; however, whether or not it would be applicable in the field need to be studied.

## Conclusions

Plant cells are subjected to series of sever abiotic stresses leading to programmed cell death (PCD). It seemed that, melatonin decreased PCD in alfalfa root meristem cells using two strategies: (1) reduced ROS generation, scavenging H_2_O_2_ and ${\text{O}}_{2}^{ - }$, and increased TTC reduction thus maintain cell viability (2) reduced caspase-3-like activity, DNA laddering and up-regulation of *BI-1*, *UCP1-UCP2* genes and downregulation of *γVPE* gene.

## Data Availability

In case of request, it would be available.

## Abbreviations

PCD: Programmed cell death
ROS: Reactive oxygen species
H_2_O_2_: Hydrogen peroxide
O_2.−_: Superoxide radical
GSH: Glutathione
ASC: Ascorbate
BI-1: Bax inhibitor-1
ER: Endoplasmic reticulum
VPE: Vacuolar processing enzymes
MPTP: Mitochondria permeability transition pore
UCP: Uncoupling proteins
MS: Murashige Skoog
TCA: Trichloroaceticacid
NBT: Nitroblue tetrazolium
DCFDA: 2′,7′-Dichlorofluorescin diacetate
DCF: 2′,7′-Dichlorofluorescein
TTC: Triphenyl tetrazolium chloride
TF: Triphenylformazan
CTAB: Cetyl trimethylammonium bromide
PVP: Polyvinylpyrrolidone
EDTA: Ethylene-diamine-tetraacetic acid
DTT: Dithiothreitol
cAMP: Cyclic adenosine monophosphate
FB1: Fumonisin B1
